# Metabolic syndrome prediction based on body composition indices

**DOI:** 10.1186/s42506-024-00181-9

**Published:** 2024-12-23

**Authors:** Buthaina Alkhatib, Aliaa Orabi, Lana M. Agraib, Islam Al-Shami

**Affiliations:** 1 Department of Clinical Nutrition and Dietetics, Faculty of Applied Medical Sciences, The Hashemite University, Zarqa, Jordan; 2 Department of Nutrition and Food Technology, Faculty of Agriculture, The University of Jordan, Amman, Jordan; 3 Department of Nutrition and Food Science, Faculty of Allied Medical Sciences, Al-Balqa Applied University, Al-Salt, Jordan; 4 Zarqa University College, Al-Balqa Applied University, Zarqa, Jordan

**Keywords:** Metabolic syndrome, Body composition, Anthropometric indicators, Fat mass percentage, Muscle mass percentage, Fat mass index

## Abstract

**Background:**

Metabolic syndrome (MetS) is an important public health issue that has been lately linked as a growing concern worldwide.

**The objective:**

To find out which anthropometric and body composition indices can prognosticate MetS in Jordanian adult females.

**Methods:**

A sample of 656 Jordanian adult females was recruited (January–March 2024) in the middle of Jordan. Weight, height, waist and hip circumference, lipid profile (triglycerides and high-density lipoprotein), fasting plasma glucose, and blood pressure were measured. Fat mass index (FMI), body mass index (BMI), fat-to-muscle ratio, and waist-to-hip ratio (WHR) were calculated. The presence or absence of MetS was the outcome of interest. Receiver operating characteristic (ROC) analyses were used to examine the predictive accuracy of the indices, and the area under the curve (AUC) was measured.

**Results:**

40.6% had MetS, and their mean age was 45.5 years. 90.2% of the participants with MetS were obese based on body fat percentage. The MetS participants had significantly higher means of all the anthropometric indices except the fat-to-muscle ratio. None of the MetS participants were underweight, and 70.8% and 73.8% were obese based on BMI and WHR, respectively (*p* < 0.001). The highest proportion of the MetS participants (35.5%) was within the Q4 of the FMI compared to those without MetS (*p*<0.001). The discrimination ability for all indices was almost equal in predicting the existence of MetS (fair prediction power; AUC = 0.66–0.72), except for the fat-to-muscle ratio, which had poor prediction power.

**Conclusion:**

Fat mass %, muscle mass %, FMI, BMI, and WHR could be used as predictors of MetS in Jordanian females, while the fat-to-muscle ratio was not. We suggested that more extensive sample size studies from both genders and different age categories are necessary to develop a superior predictor for MetS in Jordan.

## Introduction

The National Cholesterol Education Program (NCEP) Adult Treatment Panel III (ATP III) defined the metabolic syndrome (MetS) as a cluster of 5 metabolic disorders that are significantly related to cardiovascular diseases (CVD) and type 2 diabetes (T2D) development risk, as well as abdominal obesity, insulin resistance, glucose intolerance, dyslipidemia, and hypertension. Consequently, those who met three out of five of these metabolic disorders were diagnosed with MetS [1]. MetS is considered a silent epidemic with high prevalence among adults and high costs for public health systems worldwide [2]. In Jordanian adults, MetS prevalence ranged from 36% to more than 51% according to NCEP, ATP III definition [3].

Obesity, particularly abdominal obesity, is a risk factor with the highest impact on the development of MetS [4]. Body mass index (BMI), and waist circumference (WC) are used to evaluate obesity and predict MetS. As a result, they are regarded as a straightforward, inexpensive, and easy-to-measure method [2, 5]. Furthermore, it has been found that the waist-to-height ratio (WHR) index and WC had the highest power to predict MetS, and all anthropometric indices with BMI could predict the component of MetS except low HDL [6]. Even though there are restrictions to using BMI and WC as predictor indices, BMI has low precision in assessing adipose tissue distribution and age-linked body composition [7]. In addition, WC cut-off points have ethnic diversity, and the definition of obesity differs in Asia, North America, and Europe. For example, a WC of 102 cm and over for men and 88 cm for women is considered high [8]. The abovementioned limitations provoke ongoing research into novel and more valuable metrics for MetS prediction.

The incapability of BMI to discriminate between body fat and body lean mass makes it a less significant risk factor for MetS and obesity, and several studies have looked into the possible significance of body composition measurements in MetS prediction [9]. Dual-energy X-ray Absorpometry (DXA) and computed tomography (CT) are the gold standards for measuring body composition and body fat distribution [10]. However, its cost and limited convenience have made it challenging to employ in broad populations, which has limited the number of people who can potentially be screened for MetS, particularly in developing nations [11]. The most popular alternative technique for determining body composition and body fat percentage (BF%) in experimental practice is bioelectrical impedance analysis (BIA), which is characterized by being accurate, easy to use, and affordable [12].

To expand the approaches that are now mainly focused on weight loss, it is vital to understand the precise fat distribution related to MetS risk [13]. It has been proposed that BF% and fat mass index (FMI) were positively correlated to the MetS component and may be used with reasonable accuracy to predict Mets in undergraduate students [14]. A cross-sectional study indicates that MetS with high FMI against those with lower FMI appear to be independently and positively associated regardless of BMI and BF% in both sexes [15]. This study aims to determine which anthropometric and body composition indices can predict MetS in Jordanian adult females.

##  Methods

### Study design and sample

 A cross-sectional study was conducted from January to March 2024. The target sample was sourced from al-Basheer Hospital and Zarqaa Governmental Hospital, two key government hospitals in Jordan’s central regions. The participants were outpatient clinic visitors and/or caregivers who frequently visited these institutions for follow-up care. The inclusion criteria were being Jordanian, females over eighteen, able to communicate, not diagnosed with chronic diseases such as cardiovascular diseases, hypertension, and diabetes, not using hypolipidemic drugs or dietary supplementations, and willing to participate. Pregnant women and Jordanians under eighteen were excluded.

The Hashemite University’s institutional review board (No.1/7/2022/2023) and the Jordanian Ministry of Health (MOH/REC/2023/375) reviewed and approved the study protocol. All willing and eligible subjects gave their informed written agreement before being included in the study.

The Raosoft online calculator was used to determine the sample size based on a 50% response distribution, a 5% error margin, a 95% confidence interval, and an additional 10%; the minimum sample size required was 424 participants (656 female participants were included, however, 94 males were excluded due to unrepresentative sample).

### Data collection and measurements

Under the direction of the lead investigators, a certified nutritionist performed the participant interviews, obtained the required information, and verified the accuracy of the anthropometric data collected.

Seca stadiometers (Seca, USA) were used to measure body height. In contrast, the bioelectrical impedance analysis (BIA) (electrical InBody H20N body fat Scale (FSA, USA)) was used to measure weight (kg), fat mass%, and muscle mass%. Also, body mass index (BMI) (kg/m^2^) was calculated and classified. RIEDER Body Measure tape, Lock Pin, and Push-Button Retract (Inct. Bonus Kit, REIDHK, China) were used to measure waist circumference (WC) and hip circumference (HC) to the nearest 0.1 cm. Moreover, the waist-to-hip ratio (WHR) was calculated. The categorization of the subjects based on WHR was < 0.80 (normal), 0.80 to 0.84 (overweight), and ≥ 0.85 (obese) for females [16]. Based on WHO, fat mass% ≥ 35% are classified as obese for females [17]. Fat mass index (FMI) = (fat mass (kg)/(height (m)^2^); however, fat mass = fat mass% * weight (kg) [15]. FMI was categorized into quartiles as follows: Q1 < 9.58, Q2 9.58–12.65, Q3 12.65–16.4, Q4 > 16.4. The fat-to-muscle ratio was calculated as (fat mass (g)/muscle mass (g)), and was also categorized into quartiles as follows: Q1 < 1.50, Q2 1.50–1.74, Q3 1.75–1.98, Q4 > 1.98.

### Blood pressure measurements, biochemical data, and MetS determination

Concerning the hospital’s procedures, BP was assessed using a numerical blood pressure (BP) monitor (A&D Medical, UA-7675 digital blood pressure monitor, Japan). The mean of the second and third readings were used to calculate the participant's BP status. Fasting blood glucose (FBG) and serum lipids (mmol/L) were gathered from the Hakim online medical system. Regarding the National Cholesterol Education Program Adult Treatment Panel III (NCEP-ATPIII), MetS components were determined [18]. The diagnostic criteria of MetS If three or more of the following five MetS components (i.e., WC ≥ 102 cm in men and ≥ 88 cm in women, FBG ≥ 6.1 mmol/L) or use of anti-diabetic medication, blood pressure (BP); SBP ≥ 130 mmHg or DBP ≥ 85 mmHg or on BP-lowering medication, TG ≥ 1.7 mmol/L or medication for elevated TG, and HDL < 1.03 mmol/L (male) < 1.29 mmol/L (female) or taking medication for reduced HDL [19].

### Statistical analysis

The IBM SPSS Statistics (IBM SPSS Statistics for Windows, Version 23.0. Armonk, NY, USA: IBM Corp.) was used to analyze data. The presence of MetS was the critical differentiation factor among the study population. The Shapiro–Wilk test was used to verify the normality of variables. So, means with standard deviations expressed normally distributed continuous variables. However, categorical variables are presented as frequencies and percentages (%). The chi-square (*χ*2) test was performed to test the differences between participants with and without MetS. The independent *t*-test was performed to compare the means of continuous variables between the two groups. Rreceiver-operating characteristic (ROC) curve analyses were performed to determine the discriminatory power of the calculated anthropometric indices as classifiers for the presence of MetS. Following, the areas under the curve (AUCs) were calculated to compare the discriminatory power of each index. Differences among AUCs were compared to identify the best index in MetS prediction among Jordanian adult females; indices with the biggest AUC were considered the best. Excellent discrimination ability is when the AUC is between 0.9 and 1.0 while AUC from 0.80 to 0.90 indicates good, 0.70 to 0.80 indicates fair, 0.60 to 0.70 indicates poor, and 0.50 to 0.60 indicates fail discrimination ability. Youden’s *J* statistic was used to determine the optimal cut-off points for all indices using the following equation: *J* max. = Sensitivity + Specificity – 1. The index values corresponding to the maximum value of Youden’s *J* statistic were recognized as optimal cut-off points for these indices using MedCalc (22.023). Findings with a *p*-value of < 0.05 were statistically significant.

## Results

### Baseline characteristics

A sample of 656 Jordanian adult females were enrolled in this study, and the participants’ mean age was 45.5 years. Of these participants, 40.6% were having MetS. The features of the study population based on MetS status are summarized in Table 1. Based on MetS status, participants were separated into two groups: absence or presence of MetS. Almost all the participants' general characteristics significantly differed between both groups. Means of age, anthropometric measurements, circumferences, BP measurements, and biochemical tests were higher among the participants with MetS. Most of the MetS participants had an educational level at high school or lower (78.9%), were married (81.6%), physically inactive (61.3%), and 90.2% of the MetS participants were considered obese based on body fat %. Furthermore, 24.1% of MetS’s participants were smokers, did not work (41.7%), and about 44.4% were classified as passive smokers.

**Table 1:** General characteristics of the study population stratified by MetS status (*n* = 656), Jordan, 2024

Variables	Absence of MetS (*n* = 390)	Presence of MetS (*n* = 266)	*F*-value	*P*-value
	Mean ± SD			
Age (year)	39.63 ± 11.83	51.44 ± 9.39	178.79	** < **0.001
Weight (kg)	73.99 ± 17.00	84.71 ± 15.91	65.96	** < **0.001*
Height (cm)	160.12 ± 5.89	159.71 ± 6.16	0.709	0.400
Waist circumference (cm)	91.37 ± 13.80	104.69 ± 12.24	160.14	** < **0.001*
Hip circumference (cm)	108.76 ± 13.06	116.77 ± 11.81	63.73	** < **0.001*
Systolic BP	111.22 ± 14.43	129.82 ± 20.26	187.24	** < **0.001*
Diastolic BP	77.37 ± 10.84	85.68 ± 11.90	85.15	** < **0.001*
Blood glucose (mmol/L)	5.91 ± 2.65	8.79 ± 4.77	96.81	** < **0.001*
LDL (mmol/L)	3.03 ± 1.03	3.21 ± 1.02	1.99	0.159
HDL (mmol/L)	1.28 ± 0.35	1.29 ± 0.49	0.007	0.933
TG (mmol/L)	1.21 ± 0.48	2.13 ± 1.24	67.25	** < **0.001*
*N* (%)				
Educational level				
Primary and secondary school	89 (22.8)	102 (38.3)	** <** 0.001*	
High school	184 (47.2)	108 (40.6)		
Student (college/university)	69 (17.7)	25 (9.4)		
B.Sc. degree	45 (11.5)	31 (11.7)		
Higher education (master’s/Ph. D.)	3 (0.8)	0 (0.0)		
Marital status				
Married	280 (71.8)	217 (81.6)	** < **0.001*	
Single	83 (21.3)	14 (5.3)		
Divorced	17 (4.4)	8 (3.0)		
Widow	10 (2.6)	27 (10.2)		
Income				
Less than 300 JD	104 (26.7)	75 (28.2)	0.002*	
300–600 JD	130 (33.3)	70 (26.3)		
601–1000 JD	28 (7.2)	7 (2.6)		
Do not work	117 (30.0)	111 (41.7)		
Other	11 (2.8)	3 (1.1)		
Smoking				
Non-smoker	99 (25.4)	62 (23.3)	0.001*	
Smoker (cigarettes and/or hookah)	141 (36.2)	64 (24.1)		
Passive smoker	136 (34.9)	118 (44.4)		
Ex-smoker	14 (3.6)	22 (8.3)		
Physical activity				
No	245 (62.8)	163 (61.3)	** < **0.001*	
Yes	145 (37.2)	103 (38.7)		
Obesity based on body fat%				
Non-obese	105 (26.9)	26 (9.8)	** < **0.001*	
Obese	285 (73.1)	240 (90.2)		

* P-value < 0.05 considered statistically significant. *t*-test was used for continuous variables and chi-square for categorical variables, *LDL* low-density lipoprotein, *TG* triglycerides, *HDL* high-density lipoprotein, *SD* standard deviation

### Association of the anthropometric and body composition indices with MetS presence

Among the study population, MetS participants had significantly higher means of all studied indices except the fat-to-muscle ratio, as presented in Table 2. The mean of fat mass % (44.23 ± 6.35%), muscle mass % (25.69 ± 4.37%), BMI (33.40 ± 5.90 kg/m^2^), FMI (15.13 ± 4.42), and WHR (0.90 ± 0.07) were significantly higher among MetS participants compared to the participants without MetS (39.91 ± 4.83, 23.64 ± 3.91, 28.88 ± 6.35, 11.96 ± 4.82, and 0.84 ± 0.07; respectively) *p* < 0.001. Regarding the BMI categories, 70.8% of MetS participants were obese compared to 37.7% of participants without MetS (*p* < 0.001). Interestingly, while only 4% of the MetS participants were within the normal weight category, none were categorized as underweight. Significantly, the highest proportion of the MetS participants was within the Q4 of the FMI (35.5%) and the lowest within the Q1 (8.2%) compared with participants without MetS, where the highest was Q1 (36.1%) and the lowest Q4 (17.9%) (*p* < 0.001). Participants with MetS were found to be more obese by the WHR definition compared to the participants without it (73.8% vs. 46.5%; respectively, *p* < 0.001).

**Table 2:** Association between anthropometric indices and MetS among the study participants as continuous and categorical variables (*n* = 656)

Anthropometric indices	Absence of MetS	Presence of MetS	*F*-value	*p*-value *
	(*n* = 390)	(*n* = 266)		
	Mean ± SD			
Fat mass%	39.91 ± 4.83	44.23 ± 6.35	49.39	** < **0.001*
Muscle mass%	23.64 ± 3.91	25.69 ± 4.37	39.13	** < **0.001*
Body mass index (kg/m^2^)	28.88 ± 6.35	33.40 ± 5.90	79.86	** < **0.001*
FMI	11.96 ± 4.82	15.13 ± 4.42	68.07	** < **0.001*
Fat-to-muscle ratio	1.72 ± 0.40	1.76 ± 0.33	1.73	0.188
WHR	0.84 ± 0.07	0.90 ± 0.07	97.58	** <** 0.001*
*N* (%)				
Body mass index categories				
Underweight	11 (3.0)	0 (0.0)	** < **0.001*	
Normal weight	96 (26.2)	10 (4.0)		
Overweight	121 (33.1)	63 (25.2)		
Obese	138 (37.7)	177 (70.8)		
FMI categories				
Q1	133 (36.1)	20 (8.2)	** < **0.001*	
Q2	95 (25.8)	58 (23.7)		
Q3	74 (20.1)	80 (32.7)		
Q4	66 (17.9)	87 (35.5)		
WHR categories				
Normal	118 (32.7)	16 (6.3)	** < **0.001*	
Overweight	75 (20.8)	50 (19.8)		
Obese	168 (46.5)	186 (73.8)		

*SD* standard deviation, *MetS* metabolic syndrome, *WHR* the waist-to-hip ratio, *FMI* fat mass index

^*^P-value < 0.05 is considered statistically significant, *t*-test was used for continuous variables and chi-square for categorical variables

### ROC curve and cut-off points of calculated indices to predict MetS

**Fig. 1:**
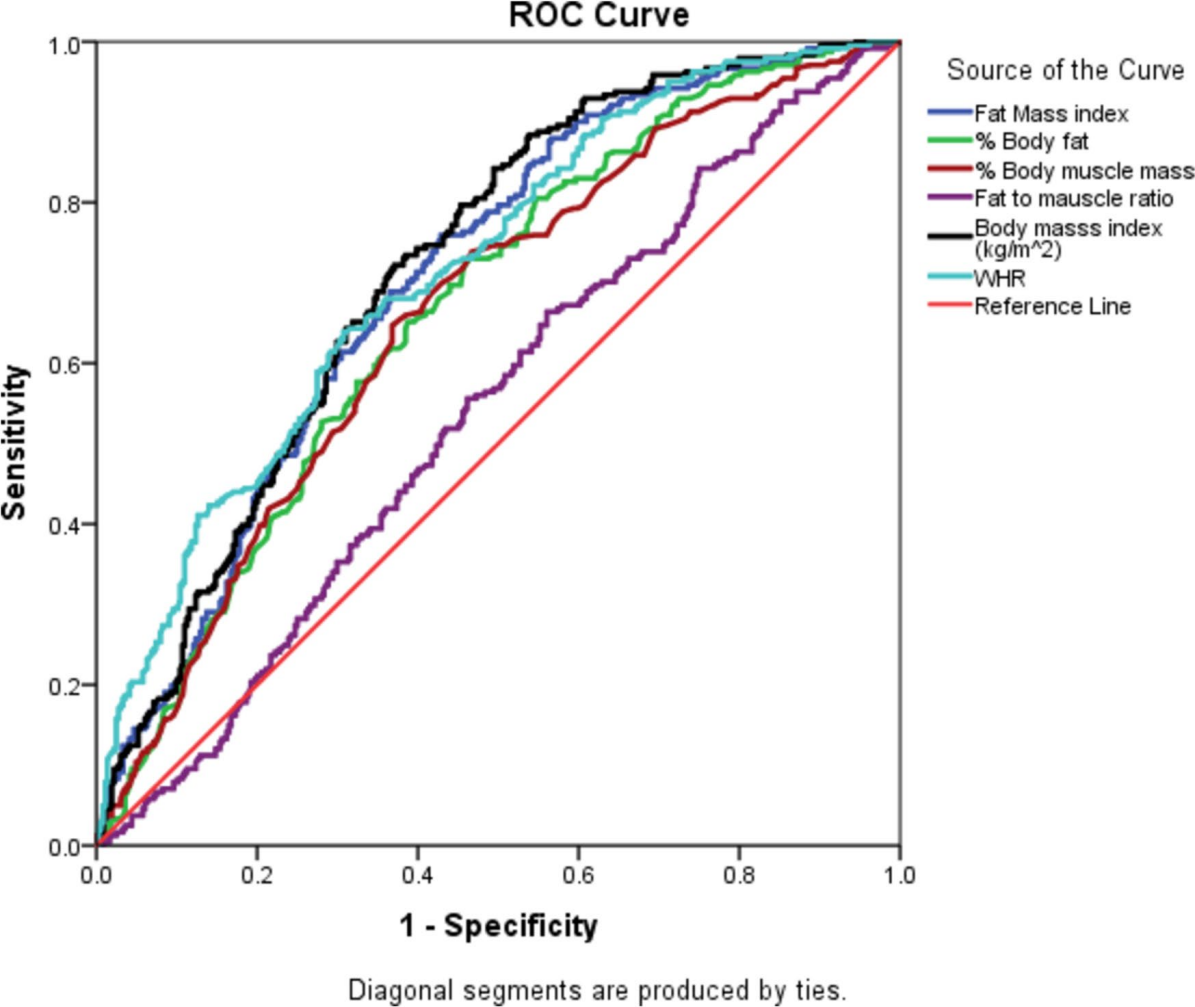
Receiver-operating characteristic curves of anthropometric measurements to identify MetS (MetS classic: three or more components out of five)

Table 3 and Fig. 1 show the area under the curve (AUC) and ROC curves comparing the predictive power of FMI, BMI, WHR, fat mass%, muscle mass%, and fat-to-muscle ratio for MetS presence among the study population. Except for the WHR, which had poor MetS discrimination ability, all anthropometric indices performed almost equally well in predicting the existence of MetS (fair MetS discrimination ability) (Table 3). Similar results were shown for fat mass% and muscle mass%, where they performed equally in predicting MetS (AUC = 0.66); for both BMI and WHR, the AUC was 0.72, while for the FMI, the AUC was 0.7. However, the AUCs of the fat-to-muscle ratio failed to have MetS discrimination ability, as shown in Fig. 1.

**Table 3:** The AUCs of the calculated anthropometric indices for the presence of MetS among the study population

Variable(s)	AUC	*P*-value	95%CI	Sensitivity	Specificity	Cut-off value	Youden Index
FMI	0.701	** < **0.001*	0.660–0.742	68.2%	62.0%	> 12.65	0.301
Fat mass%	0.664	** < **0.001*	0.621–0.706	89.5%	28.7%	> 35.0	0.182
Muscle mass %	0.657	0.043*	0.613–0.701	12.0%	92.8%	≤ 29.0	0.048
Fat-to-muscle ratio	0.525	0.283	0.485–0.564	54.4%	52.2%	≤ 1.74	0.066
Body mass index	0.715	** < **0.001*	0.675–0.755	90.6%	33.1%	> 25.0	0.236
WHR	0.717	** < **0.001*	0.676–0.758	93.2%	29.2%	> 0.80	0.225

*AUC* area under the curve, *CI* confidence interval, *WHR* waist-to-hip ratio, *FMI* fat mass index

The ability to predict the presence of MetS was almost the same for all the calculated indices; the sensitivity level was higher for WHR, BMI, and fat mass, while it was lower for mass-to-muscle and fat mass. The optimal cut-off points for predicting the presence of MetS were > 12.65 for FMI, > 35.0% for fat mass %, ≤ 29.0% for muscle mass %, > 25.0 kg/m^2^ for BMI, and > 0.80 for WHR. Meanwhile, the specificity was higher for muscle mass %, followed by FMI (Table 3).

## Discussion

Despite the large quantity of studies that have covered the topic of metabolic syndrome from many aspects and for different age groups, it is a growing and active problem for researchers. The current study aimed to determine which anthropometric and body composition indices can predict MetS in Jordanian adult females. The prevalence of MetS among studied participants was 40.6%. The prevalence was slightly lower than that reported by Ajlouni and colleagues (2020) in Jordan, whereas the crude prevalence of MetS was 48.2% (52.9% among men and 46.2% among women; *p* < 0.001) [3].

Most MetS participants in the present study had an educational level of high school or lower, were married, physically inactive, and obese based on body fat %. Furthermore, 24.1% of MetS participants were smokers, did not work (41.7%), and about 44.4% were classified as passive smokers. Many previous authors approved the aforementioned factors that affected the prevalence of MetS. Education level was negatively linked with MetS prevalence among Korean adults [20]. It has been reported that MetS patients with high education had significantly better metabolic health compared to MetS patients with lower levels. It is correlated with substantially less risk for WC, systolic blood pressure, glucose, glycosylated hemoglobin, TG, HDL, and MetS (all *p* < 0.05) in Mexico [21]. Moreover, in both sexes in Korea, the low educational group had a higher prevalence and incidence of MetS than those in the high educational group [22]. Among the adult Saudi population, it has been approved that high income [*p*] and education level inversely associated with MetS, and unemployed class was significantly positively associated with MetS [23]. Regarding physical inactivity, physical inactivity combined with high TV viewing increased the likelihood of having MetS (OR = 1.89 [95%CI 1.08–3.29]) in Brazil [24]. Also, only in Chilean adult males did there a correlation between physical inactivity with MetS (OR 1.92 [1.42–2.58], *p* < 0.01) and central obesity with MetS observed (OR=1.74 [1.23–2.47], *p* < 0.01) [25]. A study in Iran revealed that no significant changes in MetS risk *z* score were observed in single Iranian patients compared to married patients [26]. This was agreed by Jung and colleagues, who concluded that the middle-aged widowed group tends to have a higher risk of MetS compared to the middle-aged married group of Korean women, which was suggested to be associated with socioeconomic issues and health behavior [27]. In line with the present findings, among the Palestinian population in the Gaza Strip, the prevalence of MetS with age increased significantly, and it was correlated to physical activity and marital status [28]. In Iran, it has been found that age (OR= 1.06), education (OR = 0.98), and smoking (OR = 0.50) had significant effects on MetS; however, gender, marital status, and economic status had no significant impact on MetS [29].

Many studies found a high correlation between smoking (active or passive) and MetS. In a population-based study among Chinese households, it was found that adults exposed to environmental tobacco smoke > 5–7 days per week had a significantly higher risk of MetS, elevated TG, reduced HDL, and central obesity compared to those exposed to environmental tobacco smoke ≤ 4 days per week [30]. In line with the current findings, passive smoking was linked with higher levels of total cholesterol, TG, and total cholesterol: HDL ratio in comparison to individuals who were not exposed to passive smoking [31]. In contrast, from a large longitudinal cohort study, the new and continuous environmental tobacco smoke exposure groups showed an increased risk of MetS compared to the no ecological tobacco smoke exposure group, suggesting that avoidance of environmental tobacco smoke may not increase the risk of incidence of MetS [32].

Among the present study population, MetS participants had significantly higher means of fat mass %, muscle mass %, BMI, FMI, and WHR than those without MetS. In agreement, it has been found that higher FMI levels were positively associated with MetS regardless of BMI and body fat [15]. Accordingly, the number of MetS components was shown to be directly correlated with weight, BMI, fat mass, lean mass, and visceral fat area; additionally, the severity of the MetS was associated with body composition in females [33]. Additionally, body fat percentage was significantly higher in individuals with MetS and diabetes versus those without MetS and diabetes mellitus (*p* < 0.05) [34]. Additionally, body fat% and FMI were positively associated with MetS components (*p* < 0.05). Body fat% and FMI thresholds of 25.55% and 6.97 kg/m^2^ in males and 38.95% and 11.86 kg/m^2^ in females were telltale of high MetS risk [14].

The results on the association between muscle mass and the incidence of MetS were inconsistent. Kim and colleagues discovered that high-fat mass (*p* < 0.001) attenuates the protective association between muscle mass and MetS compared to low muscle/low fat in Korean adults. The prevalence of MetS was substantially correlated with low muscle/high fat (IRR 1.90; 95% CI 1.44–2.50, *p* < 0.001) and high muscle/high fat (IRR 2.30; 95% CI 1.76–3.00, *p* < 0.001) [35]. Contracting our findings, adolescents with low muscle mass had a significantly higher chance of developing MetS (OR 5.28; 95% confidence range, 2.76–10.13) even after controlling for possible confounders. Similarly, compared to people without low muscle mass, those with low muscle mass had considerably higher ORs for the MetS components [36].

As for MetS prediction ability among currently studied indices, except for the fat-to-muscle ratio, which had poor MetS discrimination ability, all anthropometric indices performed almost fair MetS discrimination ability for both BMI and WHR, the AUC was 0.72, while for the FMI, the AUC was 0.7. However, the AUCs of WHR failed to have MetS discrimination ability. Several studies indicated the MetS prediction ability for different indices. Shukohifar et al. (2022) concluded that BMI and body fat% should be considered in screening phases; body fat % could be regarded as a better predictor of hypertension and abnormalities of TG and HDL than BMI in females [37]. As revealed by the ROC curve analysis, the better indicators of MetS in women were WC and WHR parameters than BMI; WC and WHR had a higher sensitivity for predicting MetS in females than males. The cut points for WC were nearly equal in men and women, 90.3 versus 90.0, respectively. Moreover, females had higher cut points for BMI (28.5 kg/m^2^) compared to men (26.0 kg/m^2^) [38]. These findings were agreed with the current findings. Moreover, WC showed the highest AUC (0.785), so it predicted MetS more accurately than both BMI (0.733) and WHR (0.783). The highest AUC was observed in WHR (0.837), followed by WC (0.799) [39]. Among Colombia university students, body fat and FMI were positively associated with MetS components (*p* < 0.05) and may be used moderately to recognize MetS. Body fat% and FMI cut-off of 38.95% and 11.86 kg/m^2^ in women were indicators of high MetS risk [14].

Contracting the present findings, in a Korean study, the fat-to-muscle ratio was found to be a MetS predictor; the optimal cut-off value was higher in women than in men (0.555 vs. 0.336, respectively), affected by BMI; normal-weight participants (0.9992 in women and 0.9986 in men) had the highest negative predictive value, while obese participants (0.5994 in women and 0.5428 in men) had the highest positive predictive value [40]. Similarly, the Chinese study by Kim and colleagues observed that the cut-off value for the women's fat-to-muscle ratio was 0.55, and the area under the curve was 0.91 [41]. It has been noticed that MetS prediction ability for different anthropometric and body composition indices was affected by body weight, BMI classification, gender, and age group.

### Study limitations 

The study’s main strengths were its rich information resources and the many gathered and computed evaluation measures. Furthermore, all information was acquired by skilled interviewers who had received training, using reliable and valid questions. Due to the nature of cross-sectional studies, a causal relationship cannot be demonstrated. The tiny and unrepresentative male sample was excluded. Because of this, further research is necessary, especially for men, as our findings may not apply to different demographics.

## Conclusion

The prevalence of metabolic syndrome among Jordanian females was high. Body composition and anthropometric indices could be used to predict metabolic syndrome. Fat mass percentage, FMI, BMI, and WHR were the suggested predictors of MetS in Jordanian females, while the fat-to-muscle ratio was not. We suggested that more extensive sample size studies from both genders and different age categories are necessary to develop superior and accurate indices to predict MetS in Jordan.

## Data Availability

The data presented in this study are available on request from the corresponding author.
